# Effects of a Hyperbaric Normoxic Environment on the Retina and Choroid

**DOI:** 10.1167/iovs.66.3.47

**Published:** 2025-03-21

**Authors:** Nur Demir, Belma Kayhan, Yavuz Aslan

**Affiliations:** 1 Ophthalmology Department, Sultan 2. Abdulhamid Han Training and Research Hospital, University of Health Sciences, Istanbul, Turkey; 2 Undersea and Hyperbaric Medicine Department, Sultan 2. Abdulhamid Han Training and Research Hospital, University of Health Sciences, Istanbul, Turkey

**Keywords:** choroid, hyperbaric chamber, hyperbaric normoxic environment, hyperoxia, intracranial pressure, retina

## Abstract

**Purpose:**

Divers and individuals working in submarines and other underwater environments are exposed to normoxic hyperbaric conditions frequently. The aim of this study was to evaluate the effects of hyperbaric normoxia on the retina and choroid.

**Methods:**

Healthy participants with no prior diving experience were exposed to 2.4 atmospheres absolute pressure in a normoxic hyperbaric chamber (HC). Optical coherence tomography was used to measure retinal thickness, the peripapillary retinal nerve fiber layer, and choroidal thickness (CT) before and within 30 minutes after HC exposure.

**Results:**

The right eyes of 42 participants (mean age, 23.88 ± 2.85 years) were included in the study. The retinal nerve fiber layer thickness significantly decreased in the central 1-mm Early Treatment Diabetic Retinopathy Study (ETDRS) subfield after HC exposure (*P* < 0.05). The outer plexiform layer showed significant thickening in the central 1-mm ETDRS subfield (*P* < 0.05). The retinal pigment epithelium (RPE) thickness in the 3-mm ETDRS subfield significantly decreased after HC exposure (*P* < 0.01). Furthermore, nasal CT (*P* < 0.05), temporal CT (*P* < 0.05), and mean CT (*P* < 0.01) significantly increased after HC exposure.

**Conclusions:**

This study is the first in which the effects of a hyperbaric normoxic environment on the retina and choroid were examined. The observed increases in outer plexiform layer and CTs may result from elevated intracranial perfusion pressure, likely owing to increased venous pressure with unchanged cerebral arterial blood flow under hyperbaric normoxic conditions. Elevated intracranial perfusion pressure may also contribute to venous stasis in the retinochoroidal circulation, potentially explaining the structural changes observed in this study.

The human body is typically exposed to ambient air pressure (1,013.25 millibars at sea level). Even minor fluctuations in air pressure have been associated with subarachnoid hemorrhage,^1^ stroke,^2^ blood pressure variability,^3^ and myocardial infarction.^4^ Hyperbaric oxygen therapy (HBOT) is used widely to treat various diseases and its effects on the eye have been studied extensively. For example, one study revealed no significant changes in central macular thickness or choroidal thickness (CT) after 10 or 20 sessions of HBOT.^5^

In contrast, the effect of hyperbaric air has been studied in only a few prior studies,^6^^,^^7^ but never previously on the retina and choroid. Divers and individuals working in submarines or underwater environments are exposed to hyperbaric normoxic conditions frequently. Like HBOT, a hyperbaric normoxic environment increases the blood oxygen concentration by increasing gas solubility in the blood, albeit to a lesser extent.^8^ In one study, participants breathing air at 2.8 atmospheres absolute (ATA) experienced an increase in the arterial partial pressure of oxygen to 330.1 ± 22.16 mm Hg.^9^ During HBOT, pressures of 1.5 to 3.0 ATA are applied while 100% oxygen is inhaled, potentially increasing the arterial partial pressure of oxygen to as high as 2000 mm Hg.^10^

The choroid, characterized by its dense vascular network, plays a critical role in supplying oxygen and nutrients to the retina and serves as a heat stabilizer. The choroid lacks autoregulatory capacity—it is under both sympathetic and parasympathetic control—however, the retina has autoregulatory capacity.^11^ Hyperoxia has been shown to induce vasoconstriction in retinal vessels through autoregulatory mechanisms,^12^ whereas the choroid does not exhibit the same response to hyperoxia.^13^

Hyperbaric air exposure is expected to have both pressure and hyperoxia effects, although to a lesser degree than HBOT. The pressure-related effects on the eye and intracranial pressure (ICP) are likely to have a more significant impact on the retina and choroid than hyperoxia alone. The aim of this study was to evaluate the acute structural effects of increased ambient pressure on the retina and choroid using a hyperbaric chamber (HC).

## Methods

This prospective study was conducted in accordance with the guidelines of the Declaration of Helsinki. Ethical approval was obtained from the Clinical Research Ethics Committee (Approval No. 2024/13/874), and written informed consent was obtained from all participants.

A total of 42 volunteers who applied to the Hyperbaric and Underwater Medicine Clinic for a diving course training certificate were included in this study. None of the participants had prior diving experience. As a part of the certification requirements, routine blood tests and medical examinations were performed, and all participants were confirmed to be healthy.

The exclusion criteria were refractive errors exceeding ± 1 diopter, and any history of ocular surgery, uveitis, retinal or choroidal diseases, hypertension, diabetes mellitus, or vascular occlusive disease. All participants underwent a comprehensive ophthalmologic examination, including best-corrected visual acuity testing; slit-lamp biomicroscopy; intraocular pressure (IOP) measurement; dilated fundus examination; and spectral-domain optical coherence tomography (SD-OCT) imaging of the macula, peripapillary retinal nerve fiber layer (RNFL), and choroid. These examinations and measurements were repeated within 30 minutes after HC exposure.

### HC Test

Diver candidates underwent comprehensive evaluations, including detailed biochemical blood tests and complete skeletal radiographic imaging, as well as chest radiography. In addition, they were examined by specialists from multiple medical disciplines, including neurology, orthopedics and trauma surgery, pulmonary medicine, dermatology, cardiology, internal medicine, ophthalmology, psychiatry, general surgery, dentistry, otorhinolaryngology, and physical medicine and rehabilitation. If no contraindications for compression were identified during these clinical evaluations, the final examination was conducted in the HC.

**Figure 1.**
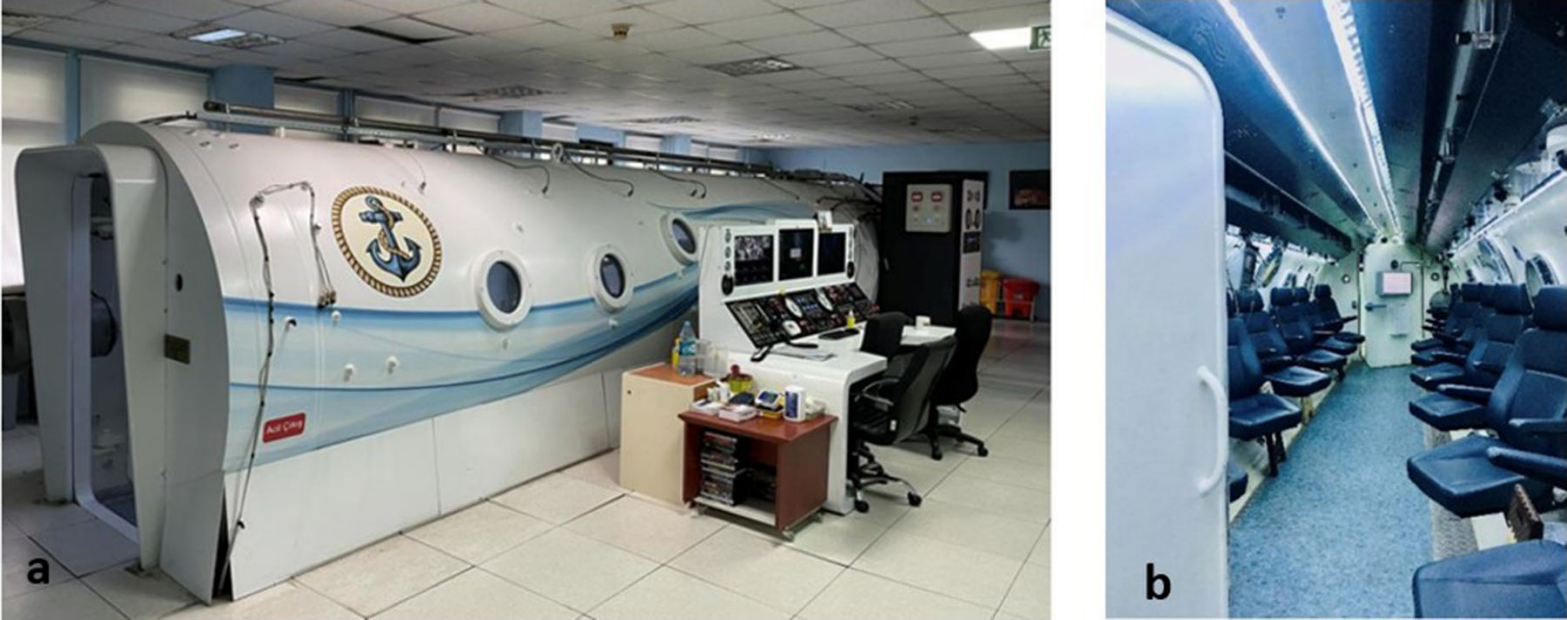
HC. (**a**) External view of the HC. (**b**) Internal view of the HC.

During the hyperbaric normoxic test, up to 12 candidates participated simultaneously in a multiplace chamber and remained seated throughout the test (Figs. 1^2^a, 1b). The compression process lasted 15 minutes, reaching a depth equivalent to 45 feet of seawater. If any candidate had difficulty tolerating the conditions at any point, they were removed to the antechamber, disqualifying them from the diver or submarine training programs.

**Figure 2.**
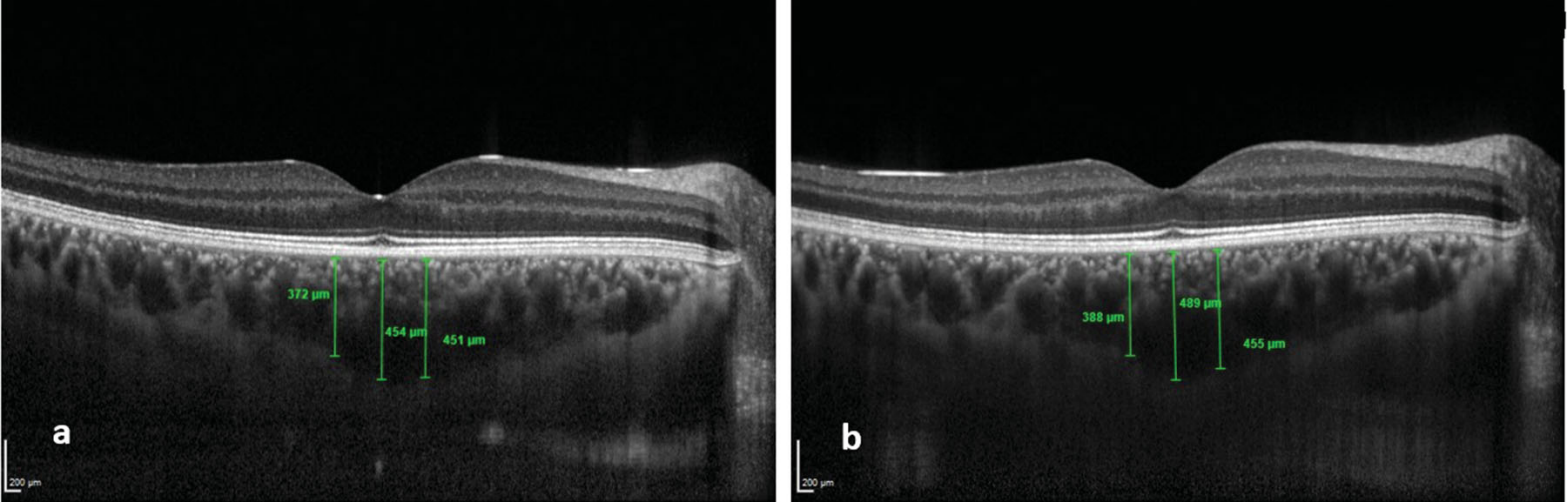
CT measurements using enhanced depth imaging optical coherence. (**a**) CT measured subfoveally and 500 µm nasal and temporal to the fovea before HC exposure. (**b**) Increased CT in the same eye after HC exposure.

After this assessment, the participants were exposed to a stable pressure of 2.4 ATA for an additional 15 minutes. The chamber was then decompressed over another 15-minute period to return to surface pressure. Compression and decompression were performed at a rate of 3 feet per minute. Candidates who completed the test without any issues were considered to have passed the test successfully.

### Retina and Choroid Examinations

The retinal and choroidal layers were measured using SD-OCT (Version 1.10.4.0, Software_V6.16.2, Heidelberg Engineering, Heidelberg, Germany). The Early Treatment Diabetic Retinopathy Study (ETDRS) grid was centered on the foveola. The total retinal thickness, along with the thicknesses of the RNFL, ganglion cell layer, inner plexiform layer, inner nuclear layer (INL), outer plexiform layer (OPL), outer nuclear layer (ONL), and retinal pigment epithelium (RPE), were segmented and analyzed across all ETDRS subfields. Peripapillary RNFL thickness in the superior, inferior, temporal, and nasal quadrants was also assessed with SD-OCT.

The choroid was imaged using enhanced depth imaging mode, and the CT was measured in the subfoveal region, as well as 500 µm temporal and nasal to the fovea, using SD-OCT. The mean of the subparafoveal measurements was calculated and used for statistical comparisons.

The OCT images were evaluated independently by two ophthalmologists at different times to ensure quality and measurement accuracy. Segmentation was performed automatically, and no manual corrections were required for any of the images.

### Statistical Analysis

The IBM SPSS 26.0 package program was used in the statistical analysis of the data. To compare all measurements before and after hyperbaric treatment, a paired-samples *t* test was used for normally distributed variables, and the Wilcoxon signed ranks test was used for nonnormally distributed variables. The Shapiro‒Wilk test was used to determine whether the variables were normally distributed. All the statistical analyses were evaluated at the 95% confidence interval**,** and a *P* value of less than 0.05 was the threshold for significance.

## Results

The right eyes of all 42 participants were evaluated. All participants were male, with a mean age of 23.88 ± 2.86 years (range, 19–40 years). The spherical equivalent, uncorrected visual acuity, and best-corrected visual acuity remained unchanged after HC exposure. None of the participants reported any visual symptoms postexposure. The descriptive characteristics of the participants are presented in Table 1. The mean IOP was 16.33 mm Hg preexposure and 15.56 mm Hg postexposure, with the difference being statistically insignificant (*P* > 0.05).

**Table 1.** Descriptive Properties of the Study Group

*N* = 42 Eyes	Mean ± SD [Min, Max]
Age (years)	23.88 ± 2.86 [19, 40]
SE (D)	0.02 ± 0.30 [*−*0.75, 0.75]
UCVA (decimal)	0.98 ± 0.07 [0.7, 1.0]
BCVA (decimal)	1.0 ± 0.0

BCVA, best-corrected visual acuity; D, diopter; SE, spherical equivalent; UCVA, uncorrected visual acuity.

RNFL thickness significantly decreased in the central 1-mm ETDRS grid subfield after HC exposure (*P* < 0.05, Table 2). No significant changes were observed in the thicknesses of the ganglion cell layer, inner plexiform layer, INL, or ONL (Table 2). The OPL exhibited significant thickening in the central 1-mm ETDRS subfield (*P* < 0.05) (Table 2). RPE thickness in the 3-mm ETDRS subfield decreased significantly after HC exposure (*P* < 0.01) (Table 2). The peripapillary RNFL thickness in all four quadrants did not significantly change after HC exposure (Table 3). The nasal CT, temporal CT, and mean CT measurements significantly increased after HC exposure (*P* < 0.05) (Table 3; Fig. 2).

**Table 2.** Comparison of Total Retinal and Individual Retinal Layer Thicknesses Before and After HC Exposure

	Before HC		After HC		
ETDRS Ring	Mean ± SD	Median	Mean ± SD	Median	*P* Value
RT					
Central 1-mm ring	268.38 ± 21.70	266	268.60 ± 21.80	266	0.613^*^
Inner 3-mm ring	348.74 ± 12.60	347.13	348.67 ± 12.60	347.13	0.813^*^
Outer 6-mm ring	304.47 ± 13.10	304.25	304.38 ± 13.50	302.75	0.828^*^
RNFL					
Central 1-mm ring	12.48 ± 2.30	12	12.00 ± 2.00	12	0.027^*^
Inner 3-mm ring	22.13 ± 1.50	22	21.96 ± 1.60	21.75	0.304^*^
Outer 6-mm ring	35.76 ± 3.80	35.63	35.45 ± 3.50	34.38	0.229^*^
GCL					
Central 1-mm ring	14.62 ± 4.40	14	14.79 ± 5.10	14	0.602^†^
Inner 3-mm ring	54.57 ± 3.80	54.38	54.23 ± 3.90	54.13	0.128^†^
Outer 6-mm ring	38.24 ± 3.00	38.38	38.02 ± 2.90	38	0.101^*^
IPL					
Central 1-mm ring	20.36 ± 3.90	20	20.29 ± 3.70	20	0.780^*^
Inner 3-mm ring	43.93 ± 2.50	43.63	44.02 ± 2.50	43.75	0.396^†^
Outer 6-mm ring	30.67 ± 2.40	30.5	31.71 ± 8.00	30.5	0.652^†^
INL					
Central 1-mm ring	17.71 ± 4.90	17	17.71 ± 5.00	17	1.00^*^
Inner 3-mm ring	41.88 ± 2.60	42.13	41.8 ± 2.90	41.5	0.766^*^
Outer 6-mm ring	34.43 ± 2.20	34.25	34.61 ± 2.30	34.5	0.134^*^
OPL					
Central 1-mm ring	21.74 ± 4.50	21	22.88 ± 5.40	22	0.033^*^
Inner 3-mm ring	31.54 ± 3.50	31.13	31.93 ± 3.30	31.25	0.224^†^
Outer 6-mm ring	27.36 ± 2.00	27.13	27.33 ± 1.90	26.75	0.988^†^
ONL					
Central 1-mm ring	93.62 ± 9.90	95	93.45 ± 9.40	97	0.807^*^
Inner 3-mm ring	72.98 ± 8.00	75	72.92 ± 8.20	74.63	0.881^*^
Outer 6-mm ring	59.11 ± 6.00	60.63	59.29 ± 6.10	60.63	0.278^†^
RPE					
Central 1-mm ring	17.12 ± 1.70	17	16.88 ± 1.70	17	0.265^†^
Inner 3-mm ring	15.60 ± 5.50	15	14.54 ± 1.50	14.25	0.009^†^
Outer 6-mm ring	13.48 ± 0.90	13.38	13.98 ± 4.00	13.5	0.353^†^

GCL, ganglion cell layer; IPL, inner plexiform layer; RT, full retinal thickness.

The provided data are in microns.

* Paired samples *t* test.

† Wilcoxon signed ranks test; *P* < 0.05; *P* < 0.01.

**Table 3.** Comparison of Choroidal and Peripapillary Nerve Fiber Layer Thicknesses Before and After HC Exposure

	Before HC		After HC		
	Mean ± SD	Median	Mean ± SD	Median	*P* Value
N-CT	397.12 ± 68.50	410.5	407.69 ± 71.60	402	0.04^*^
SFCT	418.24 ± 63.80	414.5	424.74 ± 65.30	419.5	0.055^*^
T-CT	389.60 ± 63.60	394.5	399.21 ± 66.00	395	0.023^*^
Mean CT	401.66 ± 60.10	407.5	410.55 ± 61.90	410.33	0.001^*^
S-RNFL	127.45 ± 13.60	127	127.29 ± 13.20	126.5	0.835^*^
I-RNFL	127.00 ± 14.10	127	125.98 ± 14.30	125.5	0.096^*^
N-RNFL	75.07 ± 13.20	73.5	74.43 ± 12.80	74.5	0.252^*^
T-RNFL	72.55 ± 9.50	70.5	73.50 ± 9.80	70.5	0.06^†^

I-RNFL, inferior RNFL; N-CT, nasal CT; N-RNFL, nasal RNFL; SFCT, subfoveal CT; S-RNFL, superior RNFL; T-CT, temporal CT; T-RNFL, temporal RNFL.

The provided data are in microns.

* Paired samples *t* test.

† Wilcoxon signed ranks test; *P* < 0.05; *P* < 0.01.

## Discussion

The primary finding of this study was that the 2.4 ATA hyperbaric normoxic test resulted in increases in choroidal and OPL thicknesses within 30 minutes after the test. Additional findings included thinning of the RNFL and RPE layers.

The CT increased in the nasal, temporal, and subfoveal regions, with statistically significant changes in the nasal and temporal areas. These findings suggest that hyperbaric normoxic environments affect the intracranial circulatory system, leading to elevated ICP and choroidal congestion through several mechanisms. The primary mechanism could be an increase in central venous pressure under hyperbaric conditions. The central venous pressure has been observed to increase with water depth, showing a positive correlation with water level.^14^ This increase in central venous pressure may also contribute to an increase in intracranial venous pressure.^14^ Elevated jugular venous pressure increases dural pressure, thereby increasing the ICP.^15^

Another potential explanation for increased cerebral venous pressure is internal jugular vein collapse. In the upright position, the internal jugular vein has been shown to collapse to compensate for a decrease in ICP.^15^ Additionally, jugular venous compression or vein kinking owing to head turning has been shown to increase cerebral venous engorgement.^16^

A possible additional mechanism associated with circulatory abnormalities is endothelial dysfunction.^17^ Hyperoxia-induced oxidative stress, circulating bubbles, and gas emboli have all been implicated as contributing factors.^8^ Diving affects both microcirculatory and macrocirculatory endothelial function.^17^ A study of compressed air workers and divers revealed an increased prevalence of brain lesions on magnetic resonance imaging, indicating that gas embolism alone does not explain these findings fully and suggesting the involvement of additional mechanisms.^18^ Yates^19^ raised two key considerations regarding brain lesions in divers. First, what appear as white matter lesions or lacunar infarcts on magnetic resonance imaging may, in fact, be cerebrospinal fluid–filled perivascular spaces misinterpreted as lacunar infarcts.^19^ Second, direct disruption of local vasomotor control and inadequate redistribution of cerebral blood flow may contribute to the formation of these lesions.^19^

A third contributing factor to increased intracranial perfusion pressure in a hyperbaric environment is the maintenance of steady intracranial arterial blood flow despite venous overload. Omae et al.^20^ investigated middle cerebral artery flow velocity under different ATA levels with air and oxygen supply. The middle cerebral artery flow velocity was 65 ± 15 cm/s at 1 ATA air and 61 ± 13 cm/s at 2 ATA air, but decreased to 50 ± 13 cm/s at 2 ATA O_2_. The authors concluded that hyperoxemia, rather than increased ambient pressure, decreases cerebral blood flow.^20^ Similarly, a scuba diving study revealed only subtle cerebral blood flow alterations after the dive.^21^

The impact of increased intracranial venous pressure and congestion on the choroid can be understood through its anatomical characteristics. Choroidal lobules drain blood into large choroidal veins, which are arranged in a parallel configuration before merging into the vortex vein ampullae.^22^ The superior vortex veins and central retinal vein drain into the superior ophthalmic vein, while the inferior vortex veins drain into the inferior ophthalmic vein.^22^ Both the superior and inferior ophthalmic veins ultimately drain into the cavernous sinus and pterygoid venous plexus.^22^ In the brain, arachnoid granulations facilitate cerebrospinal fluid drainage from the subarachnoid space into the venous sinuses, driven by a pressure gradient of 3 to 5 mm Hg.^23^ Increased venous pressure can subsequently increase the ICP.^23^ Li et al.^24^ reported choroidal thickening in a patient with right heart failure and attributed this thickening to choroidal venous stasis. Additionally, peripapillary choroidal thickening has been observed in patients with intracranial hypertension and is associated with impaired cerebral venous drainage and choroidal congestion.^25^ A study by Ozdemir and Çevik^26^ revealed that subfoveal CT is correlated with an increased ICP.

Environmental pressure fluctuations significantly affect ICP. Herbowski^27^ reported a positive correlation between external atmospheric pressure and ICP, suggesting that increasing ambient pressure elevates ICP directly. Although a diving environment is simulated in HCs, there is limited research in which ICP changes in divers are assessed directly. One study revealed an increased optic nerve sheath diameter in scuba divers compared with nondivers, suggesting a possible marker of elevated ICP.^28^

Hyperbaric normoxic environment does not involve high oxygen inhalation; however, the hyperbaric effect increases oxygen solubility in the blood, leading to hyperoxemia under normoxic conditions.^9^ Although hyperoxemia can cause vasoconstriction in retinal capillaries, study findings indicate that hyperoxemia does not induce vasoconstriction in the choroid.^13^^,^^29^^,^^30^

RNFL thickness significantly decreased in the 1-mm ETDRS grid subfield in the present study. This thinning may be attributed to vasoconstriction in the superficial vascular plexus in response to mildly increased arterial oxygen concentrations. A 10% increase in the blood oxygen concentration has been shown to decrease retinal blood flow by 12% to 13%, with reductions reaching 35% to 50% under hyperoxic conditions.^31^ Additionally, an OCT angiography study revealed that the most pronounced decrease in vessel density in response to oxygen and carbon dioxide concentration changes occurred in the deep retinal layer, followed by the superficial retinal layer.^32^

Another notable finding of this study was the thickening of the OPL in the central 1-mm ETDRS ring. The retina contains three vascular plexuses: the superficial capillary plexus, intermediate capillary plexus, and deep capillary plexus (DCP).^33^ An et al.^33^ used high-resolution confocal microscopy to study parafoveal plexuses and demonstrated that the superficial capillary plexus and intermediate capillary plexus have both arterial and venous connections, with the intermediate capillary plexus supplying the DCP through small arterioles. A distinctive feature of the DCP is its direct drainage into the retinal vein.^33^ The DCP is anatomically located between the INL and the OPL.^34^ Consistent with this finding, cystoid edema most commonly affects the OPL and INL, as shown by three-dimensional OCT imaging in cases of retinal vein occlusion.^35^ The OPL thickening observed in this study may similarly be attributed to venous stasis, paralleling the mechanism of choroidal thickening.

Considering the results of our study, RPE thickness significantly decreased in the 3-mm ETDRS zone. Although a decrease was also observed in the central 1-mm zone after HC exposure, the difference did not attain statistical significance. Similarly, ONL thickness decreased in both the 1-mm and 3-mm zones, paralleling the RPE changes, although this decrease was not statistically significant. The OPL, in contrast, exhibited statistically significant thickening in the central 1-mm zone and nonsignificant thickening in the 3-mm zone. The CT was found to be increased across all measured regions, including the central zone, with the parafoveal region demonstrating significantly more thickening than the other zones. Taken together, these findings suggest that the thickening of the choroid and OPL in the central 3-mm area is associated with venous stasis. At the same time, the RPE and ONL show concurrent thinning. This pattern suggests that bidirectional mechanical compression plays a role in RPE and ONL thinning. The compression likely results from the thickened choroid and OPL exerting pressure from both sides, contributing to structural changes in these layers. In a study conducted by Szalai et al.,^36^ reversible thinning was observed in retinal layers containing cell bodies, including the RPE layer, after exercise. The authors attributed this phenomenon to mechanical compression caused by increased IOP, because the outer layers lack vascular elements. The findings of Szalai et al.^36^ support our hypothesis that the RPE thinning observed in our study resulted from bidirectional compression owing to a thickened OPL and choroid, which was potentially associated with venous stasis.

Studies in which the effects of hyperoxia on the optic nerve were evaluated have demonstrated a significant decrease in optic nerve head blood flow, as measured by laser Doppler flowmetry, and in the peripapillary area, as observed with OCT angiography.^37^^,^^38^ In the present study, no changes in peripapillary RNFL thickness were observed. On the basis of our hypothesis, increased ICP should have caused thickening of the RNFL. However, a counteracting response to mild hyperoxia may have contributed to the unchanged peripapillary RNFL measurements.

In conclusion, this study is the first in which the effects of a hyperbaric normoxic environment on the retina and choroid were evaluated. The observed increase in OPL and CT may result from elevated intracranial perfusion pressure, primarily owing to increased venous pressure and unaltered cerebral arterial blood flow. Additionally, venous stasis in the retinochoroidal circulation may further have contributed to the observed structural changes. This study offers new insights into unexplained findings in the neurological and ophthalmological studies of divers. Further research on choroidal and intracranial hemodynamics in hyperbaric normoxic environments may help confirm this hypothesis.
